# DNA-Assisted Solubilization of Carbon Nanotubes and Construction of DNA-MWCNT Cross-Linked Hybrid Hydrogels

**DOI:** 10.3390/nano5010270

**Published:** 2015-03-03

**Authors:** Anatoly Zinchenko, Yosuke Taki, Vladimir G. Sergeyev, Shizuaki Murata

**Affiliations:** 1 Graduate School of Environmental Studies, Nagoya University, Furo-cho, Chikusa-ku, Nagoya 464-8601, Japan; E-Mails: vegetable.day.831@gmail.com (Y.T.); murata@urban.env.nagoya-u.ac.jp (S.M.); 2 Department of Polymer Science, Faculty of Chemistry, Moscow State University, Moscow 119899, Russia; E-Mail: sergeyev@genebee.msu.ru

**Keywords:** DNA, hydrogel, carbon nanotube, multi-walled carbon nanotubes (MWCNT), hybrid hydrogel

## Abstract

A simple method for preparation of DNA-carbon nanotubes hybrid hydrogel based on a two-step procedure including: (i) solubilization of multi-walled carbon nanotubes (MWCNT) in aqueous solution of DNA, and (ii) chemical cross-linking between solubilized MWCNT via adsorbed DNA and free DNA by ethylene glycol diglycidyl ether is reported. We show that there exists a critical concentration of MWCNT below which a homogeneous dispersion of MWCNT in hybrid hydrogel can be achieved, while at higher concentrations of MWCNT the aggregation of MWCNT inside hydrogel occurs. The strengthening effect of carbon nanotube in the process of hydrogel shrinking in solutions with high salt concentration was demonstrated and significant passivation of MWCNT adsorption properties towards low-molecular-weight aromatic binders due to DNA adsorption on MWCNT surface was revealed.

## 1. Introduction

DNA, which was studied for many years mostly as a central component of cell responsible for storage of genetic information, has, in the past decade, been broadly recognized as a molecule with a set of unique properties that can be successfully utilized in field of materials science [1,2,3]. The variety of DNA-based materials spread dramatically from simple one-dimensional DNA-templated nanowires [4] and arrays of nanoparticles [5] to more complex two- [6] and three-dimensional [7] “DNA origami” structures [8] and hybrid nanostructures supported by these DNA scaffolds [9,10].

DNA hydrogel is a relatively new type of DNA-based material promising as rare metal adsorbent [11], reactor for synthesis of metal nanomaterials [12], supercapacitor [13], vehicle for drug/gene delivery [14], and in other applications. Further functionalization of DNA hydrogels opens up new opportunities to use such hybrids for various materials science and biological applications. Accompanied by the recent progress in synthesis and application of carbon nanomaterials such as fullerenes, carbon nanotubes, and graphenes, hybrid materials incorporating these carbon nanostructure are being actively investigated, and several recent studies reported the construction and interesting properties of DNA hydrogels functionalized with carbon nanomaterials [15,16,17,18]. DNA in these studies was used due to its exceptionally efficient adsorption on the surface of carbon nanomaterials powered by self-recognition and self-organization into three-dimensional networks. Liu *et al.* [15] reported an elegant preparation of DNA and single-walled carbon nanotubes (SWCNT) hybrid hydrogel by adsorption of ssDNA on nanotubes and cross-linking via complementary DNA fragments outgoing from nanotubes, and showed that the strength of such hydrogels could be tuned by changing pH. Later, a similar assembly approach was used by Shi *et al.* [16] to construct multifunctional hydrogels of DNA and graphene characterized by a high mechanical strength and self-healing properties. These hybrid materials are promising candidates for cardiac constructs and bio-actuators [18] as well as for electrochemical energy storage devices [17]. All the above DNA-hydrogel-based systems, however, utilized sequence-programmed oligonucleotides for cross-linking, and, although these hybrid materials possess a number of outstanding properties, production of such hybrids beyond the laboratory scale is timely and costly.

On the other hand, DNA became available during the past decade as a resource obtained from waste products of fishery industry (salmon milt) and was shown to be suitable in applications required large quantities of DNA [19,20,21]. Therefore, the DNA extracted from salmon milt can be a good alternative component of a DNA hydrogel matrix for nanotubes embedment. Herein, we report a method for preparation of DNA–MWCNT hydrogels by chemical cross-linking between DNA adsorbed on MWCNT and free DNA in solution and highlight some of its properties.

## 2. Results and Discussion

### 2.1. Dispersion of MWCNT in Water in the Presence of DNA

**Scheme 1:**
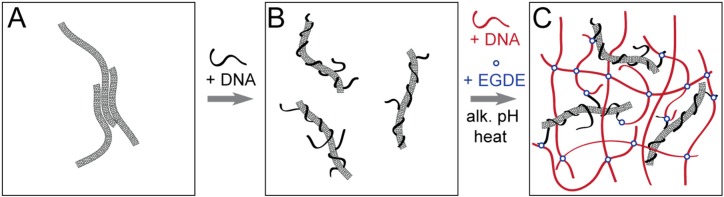
Schematic illustration of DNA–multi-walled carbon nanotubes (MWCNT) hybrid hydrogel preparation procedure. (**A**) Bundles of carbon nanotubes; (**B**) Individual nanotubes dispersed in solution due to solubilization by DNA under sonication; and (**C**) DNA-modified nanotubes cross-linked with an additionally added free DNA (red) using EGDE cross-linking agent at alkaline pH and elevated temperatures.

Construction of DNA–MWCNT hybrid hydrogel was performed as illustrated in Scheme 1. First, carbon nanotubes powder (Scheme 1A) was dispersed in aqueous solution in the presence of DNA (Scheme 1B), and then DNA-modified MWCNT were chemically cross-linked together with additionally added DNA by ethylene glycol diglycidyl ether (EGDE) to form the hybrid hydrogel (Scheme 1C).

**Figure 1:**
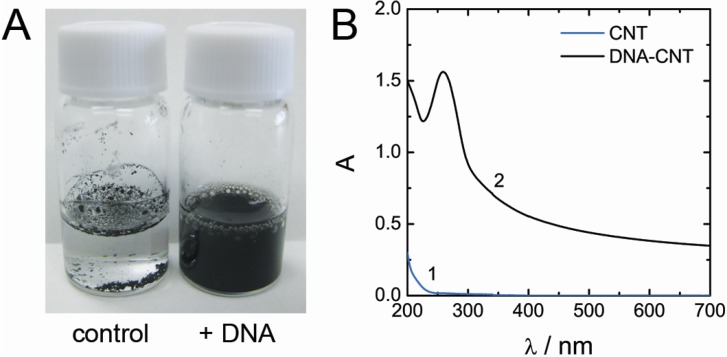
(**A**) Photographic images of MWCNT (1 mg) after dispersion in pure water (control) and in solution of 0.33% (*w*/*w*) DNA (300 bp) by sonication at 10 W for 2.5 h; (**B**) UV-Vis absorbance spectra of MWCNT aqueous solutions after dispersion by sonication in water (line **1**) and in 0.33% DNA solution (line **2**).

DNA extracted from salmon milt was used as a solubilization agent to prepare aqueous dispersions of MWCNT. Relatively short *ca.* 300 bp DNA (*ca.* 100 nm average length) was chosen to avoid deposition of single DNAs on multiple carbon nanotubes resulting in the formation of MWCNT bundles. Figure 1A shows photographic images of MWCNT in pure water (control) and in solution of 0.33% DNA after sonication. Sonication of MWCNT in pure water had no effect on MWCNT dispersion, but in the presence of DNA the solution of MWCNT after sonication turned to an intensively colored black solution indicating a good solubilization of MWCNT.

Thus prepared samples were centrifuged at 11,000 rpm for 30 min to remove insoluble MWCNT aggregates and UV-vis spectra of the resulted samples were recorded. In the absence of DNA, no absorbance of aqueous phase was detected (line 1 in Figure 1B), while the spectrum of MWCNT dispersions obtained by DNA-assisted MWCNT solubilization (line 2 in Figure 1B) had a characteristic for MWCNT broad absorbance in a visible range of spectrum indicating good solubilization of MWCNT. The peak at *ca.* 260 nm is the DNA absorbance signal. The extinction coefficient of dispersed MWCNT at 500 nm was determined by gravimetric analysis and was equal to 66 L·g^−1^·cm^−1^.

**Figure 2:**
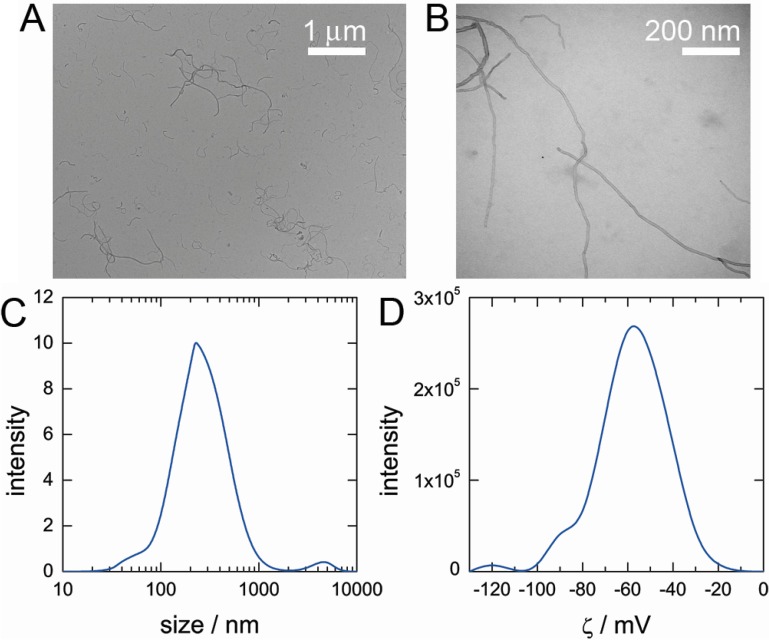
(**A**,**B**) Typical transmission electron microscopy images of MWCNT dispersed in solution of DNA. (**C**) Dynamic light scattering analysis data of MWCNT size dispersed in solution of DNA. (**D**) Zeta potential analysis data of a charge on MWCNT dispersed in solution of DNA.

Next, we utilized transmission electron microscopy (TEM) to visualize the dispersed MWCNT by DNA. TEM images in Figure 2A,B show that after sonication all individual carbon nanotubes were well dispersed and no bundles or large aggregates were present in the sample. Direct visualization of DNA adsorption on the surface of MWCNT by TEM is difficult due to weak contrast of DNA molecule and such visualization is still a challenge [22,23]. Size of nanotubes was analyzed by dynamic light scattering (DLS) (Figure 2C) and it was found that most of the nanotubes are in the range 100 nm–1 μm. The average size value was smaller than the size of original nanotubes provided by manufacturer (*ca.* 1.5 μm) due to, probably, the damage of MWCNT during sonication. Zeta potential analysis of MWCNT dispersions revealed a high negative charge on nanotubes (ζ = − 56 ± 12 mV) (Figure 2D). The high negative charge of nanotubes indicates that a large amount of anionic DNA is adsorbed on MWCNT surface.

Next, we studied the efficiency of MWCNT dispersion under various experimental conditions in order to obtain concentrated solutions of MWCNT to be further utilized for DNA–MWCNT hydrogel preparation. In every case, unless otherwise mentioned, 1 mg of MWCNT was dispersed in aqueous solution containing 0.33% DNA by ultrasound sonication and, after removal of insoluble MWCNT fraction by centrifugation at 11,000 for 30 min, the concentration of dispersed MWCNT was measured spectroscopically using molar adsorption coefficient (ε_λ = 500_) 66 L·g^−1^·cm^−1^. Equilibration of MWCNT powder with a small amount of water (20 μL of water for 1 mg of MWCNT) for 1–2 days before sonication was important to assure good dispersion ratios after sonication.

Table 1 summaries the results of MWCNT dispersion efficiency as a percentage of MWCNT amount to the original amount of sonicated MWCNT and absolute concentration of MWCNT in solutions obtained under various experimental conditions.

**Table 1:** Efficiency of 1 mg MWCNT dispersion in 3 mL of 0.33% of DNA solution under different conditions presented as an absolute concentration of MWCNT in dispersion (*c*) and as percentage of dispersed carbon nanotube to its initial amount (*m*/*m*_0_); influence of (**A**) sonication time; (**B**) solution alkalinity and temperature; (**C**) concentration of NaOH; and (**D**) the amount of MWCNT.

Sample	*c* (mg/L)	*m*/*m*_0_ (%)
**A. Sonication time**		
0.5 h	6.7	2.0
2.5 h	34.4	10.3
24 h	33.0	9.9
**B. Alkalinity and temperature**		
Room temperature, water	30.6	9.2
90 °C, water	25.4	7.6
Room temperature, 5 mM NaOH	34.4	10.3
**C. Concentration of NaOH**		
5 mM	30.6	9.2
50 mM	34.4	10.3
500 mM	5.5	1.7
**D. MWCNT amount**		
1 mg	34.4	10.3
10 mg	182.7	5.4

First, dispersion of MWCNT at various sonication times was studied (Table 1A). The increase of sonication time from 0.5 to 2.5 h resulted in increase of dispersed MWCNT concentration from 7 to 35 mg/L, while longer sonication times had no enhancement effect toward MWCNT dispersion in solution. Beside purely mechanical reason for dispersion of MWCNT that are electrostatically charged after DNA absorbance, it was also found by TEM observations that long-time sonication of MWCNT is accompanied by a shortening of MWCNT length due to MWCNT fractures, which can be an another reason for a better dispersion. Because long-time sonication increases the percentage of dispersed MWCNT but, at the same time, shortens MWCNT averages length, the appropriate sonication time has to be chosen in each particular application.

It was originally found that carbon nanotubes can be efficiently dispersed in the presence of single-stranded DNA (ssDNA) [24]. ssDNA state is considered important in contrast to dsDNA for dispersion of carbon nanotubes due to accessibility of DNA’s aromatic bases, having a high affinity to MWCNT surface because of hydrophobic interaction and π–π stacking interactions [25]. More recent studies have also revealed the interaction between dsDNA and carbon nanotubes that proceeds through dsDNA denaturation and adsorption of thus formed ssDNA on nanotube surface [26]. Taking into account the importance of DNA secondary structure for MWCNT dispersion, next, the influence of conditions promoting DNA denaturation was investigated. Table 1B summarizes the influence of elevated temperatures (90 °C) and alkaline condition as well as their combination on the efficiency of MWCNT dispersion. DNA from salmon milt used in our study contains about 65% of double-stranded and about 35% of single-stranded DNA; therefore, due to its partially denaturated character, it can absorb on a surface of MWCNT even under ambient conditions: sonication of MWCNT in 0.33% DNA solution at room temperature and at neutral pH results in about 30 mg/L dispersion efficiency.

Under conditions that cause DNA denaturation, *i.e.*, in solution with a high concentration of alkaline or in solution at 90 °C, no significant changes were observed: *ca.* 25.4 mg/L and 34.4 mg/L of MWCNT were dispersed, respectively. The combination of both, high pH and high temperature, conditions resulted in a significant decrease of dispersion efficiency down to only 8.4 mg/L. Comparison of the influence of conditions affecting DNA denaturation on the dispersion efficiency of MWCNT indicates that a high dispersion efficiency of MWCNT by DNA containing large fractions of single stranded DNA can be achieved even under ambient conditions. This can be explained by a high concentration of single–stranded DNA fragments that play the determinant role in DNA adsorption of the surface of MWCNT, thus promoting good MWCNT dispersion.

Dispersion efficiency of MWCNT was also studied upon increase in the concentration of NaOH (Table 1C). At 50 mM concentration of NaOH, slight enhancement of MWCNT dispersion efficiency was measured, while at 500 mM NaOH in solution a dramatic decrease of dispersed MWCNT was detected. This decrease is ascribed to strong electrostatic screening of DNA charges on nanotubes resulted in lowering of the colloidal stability of the nanotubes.

Finally, in order to scale up the amount of dispersed MWCNT, 10 mg of nanotubes were dispersed using the above procedure and it was found (Table 1D) that the absolute amount of dispersed MWCNT increased from 34.4 to 182.7 mg/L but relative dispersion efficiency decreased from 10.3% to 5.4%.

Although high dispersion efficiencies of carbon nanotubes were also achieved by cationic copolymers and amphiphylic peptides [27], the advantages to use DNA for the purpose of nanotube dispersion are: (i) formation of very stable complexes with MWCNT due to π–π interaction; (ii) high dispersion efficiency of MWCNT on the order of mg/mL and high stability; and (iii) availability of DNA from industrial waste products that renders DNA as green and sustainable material.

### 2.2. Preparation of DNA–MWCNT Hybrid Hydrogel

Synthesis of DNA hydrogel cross-linked by EGDE (ethyleneglycol diglycidyl ether) under alkaline conditions was first reported by Tanaka *et al.* [28], who studied physic–chemical properties of DNA hydrogel. DNA was cross-linked by a reaction between EGDE’s epoxy groups and amine groups of DNA [29] under conditions facilitating DNA denaturation, *i.e.*, alkaline pH and elevated temperatures as we described earlier.

Irregular adsorption of DNA on the surface of MWCNT as well as the presence of dsDNA fragments with a poor adsorption properties suggests that DNA-modified MWCNTs (Section 2.1) possess outgoing DNA chains on their surface as shown in Figure 1B; therefore, such nanotubes can be further co-cross-linked with a free DNA in solution using the same cross-linking agent described for preparation of DNA hydrogels [28]. To prepare DNA–MWCNT hybrid hydrogel, equal 2.5 mL volumes of 1% high-molecular mass DNA (20,000 bp) and MWCNT dispersion with a concentration of MWCNT in a range 14–110 mg/L were combined and then 15 μL of EGDE, 50 μL of 0.5 M NaOH, and 5 μL of TMEDA were successively added and thoroughly mixed. Cross-linking of MWCNT with short DNAs (about 300 bp) used for MWCNT dispersion was not efficient and gelation did not occur, therefore, highly polymerized *ca.* 20,000 bp DNA extracted from salmon milt DNA was used for the hybrid hydrogel construction. The reaction mixture was subjected to the cross-linking reaction at 90 °C and a series of DNA–MWCNT hybrid hydrogels with a varied content of MWCNT was prepared.

**Figure 3:**
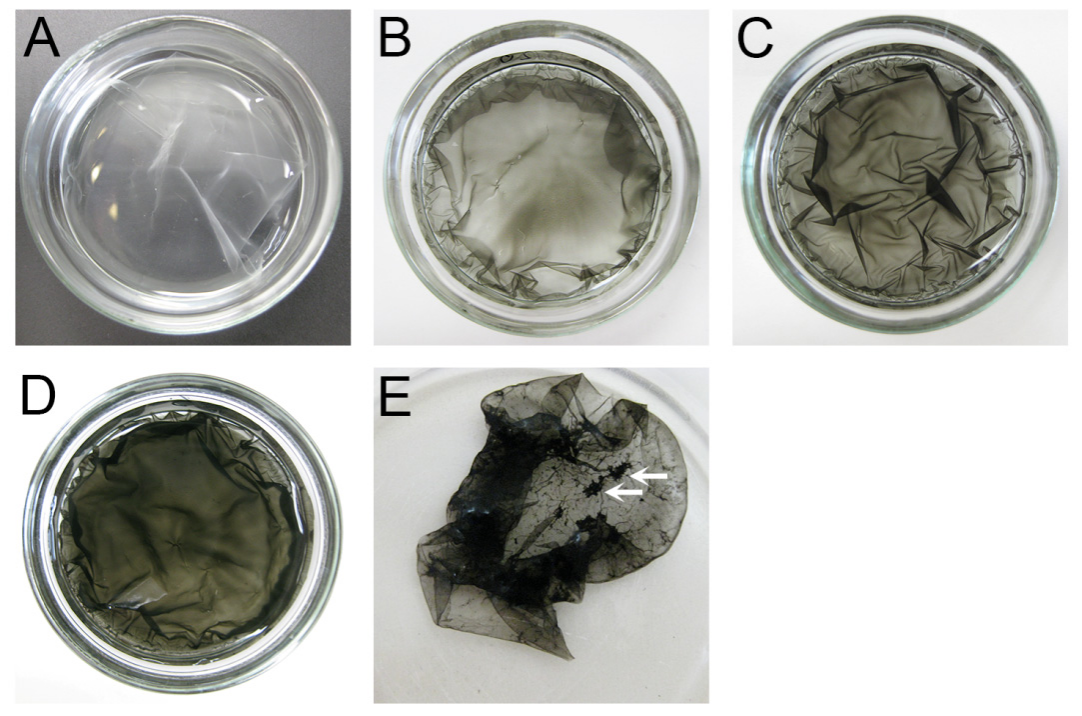
Photographic images of DNA and DNA–MWCNT hybrid hydrogel films prepared at different concentrations of MWCNT in solution (**A**: 0 mg/L (DNA hydrogel without nanotubes); **B**: 14 mg/L; **C**: 28 mg/L; **D**: 55 mg/L; **E**: 110 mg/L) after swelling in 1 mM NaCl solution. White arrows on image E indicate the flocks of aggregated nanotubes.

Figure 3A shows DNA hydrogel prepared without adding MWCNT, which appears as a transparent film containing *ca.* 3% of DNA after swelling in 1 mM NaCl solution. Figure 3B–E show photographic images of DNA–MWCNT hybrid hydrogels prepared following the same protocol by changing only the concentration of MWCNT. With the increase of MWCNT concentration from 14 to 55 mg/L the black color of the resulted hydrogel became more intense, but all the hydrogels appeared homogenous indicating good dispersion of MWCNT inside the hydrogels. Hybrid hydrogels containing different amount of MWCNT are characterized by similar swelling degrees to the original DNA hydrogels, *i.e.*, *ca.* 3% DNA contents inside hydrogel. When the concentration of MWCNT was 110 mg/L, the aggregation of MWCNT during cross-linking process inside hydrogel was observed as a formation of large black aggregated regions indicated by white arrows in Figure 3E accompanied by weakening of the overall hydrogel’s color intensity. It should be mentioned that even at the highest concentration of the MWCNT used when the homogeneous hydrogel was prepared (55 mg/L), the concentration of MWCNT in hydrogel was about 100 times lower than that of DNA.

The above results show that there is a critical concentration of MWCNT at which MWCNT can be cross-linked provided that the nanomaterials are homogeneously dispersed inside the hydrogel. Due to high concentrations of DNA counter-ions and NaOH electrolyte in the reaction mixture, stabilization of DNA–MWCNT complexes becomes not efficient at high MWCNT concentration and triggers bundling and aggregation of MWCNT in the cross-linking hydrogel. Segregation of MWCNT due to depletion in concentrated solutions of DNA can be another possible driving force.

### 2.3. Effect of MWCNT Embedment on Strength of Hybrid Hydrogels

MWCNTs with persistence lengths exceeding several hundred nanometers possess high mechanic rigidity in comparison to DNA of *ca.* 50 nm persistence lengths for dsDNA and several nm for ssDNA. Therefore, blending of MWCNT rigid scaffolds into DNA hydrogel film via chemical cross-linking is generally assumed to result in strengthening of the hybrid hydrogel. To test the influence of MWCNT on DNA hydrogel mechanical properties, we studied the shrinking of DNA hydrogel with different MWCNT contents in solutions with various concentration of low-molecular salt, NaCl.

**Figure 4:**
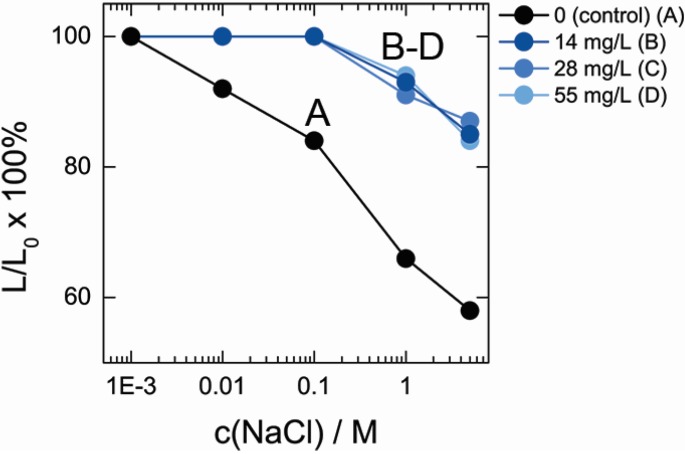
Relative decrease of DNA and DNA–MWCNT hybrid hydrogel film size in solutions of varied NaCl concentrations.

DNA hydrogel matrix represents a highly charged polyelectrolyte network, which shrinks at high ionic strengths due to electrostatic screening of DNA phosphates by Na^+^ counter-ions. Figure 4 shows the change of DNA and DNA–MWCNT hydrogel film size upon increase of the NaCl concentration in solution. When concentration of NaCl increased from 1 mM to 5 M, more than 40% decrease of MWCNT-free DNA hydrogel size was observed, which corresponds to *ca.* 5-fold decrease of hydrogel volume. Embedment of MWCNT into hydrogel matrix had a drastic effect on the shrinking of hydrogel. In contrast, an increase in salt concentration from 1 mM to 0.1 M had essentially no effect on the swelling degree of hybrid hydrogel. At higher concentrations of NaCl, shrinking of hybrid hydrogel occurred but even at the highest 5 M NaCl concentrations only 15% decrease of size was found. Noteworthy, while there was drastic effect of MWCNT on DNA hydrogel shrinking behavior, shrinking profiles of the hydrogels containing different MWCNT concentrations were very similar.

Shrinking experiment clearly indicates that hydrogels containing co-cross-linked MWCNT are very resistant to the shrinking induced by the increase of the ionic strength than the original DNA matrix and in a good agreement with earlier studies that reported the enhancement of hydrogel strength by MWCNT [15].

### 2.4. Adsorption Properties of MWCNT in DNA–MWCNT Hybrid Hydrogel

Hydrophobicity and large surface area of carbon nanomaterials renders them as good adsorbents for organic molecules, and this property of carbon nanotubes has been used for environmental pollution management [30,31]. Application of bare carbon nanotubes, however, is problematic because of the issues related to their environmental and heath toxicity [32] and suitable templates for their embedment are demanded. As shown above, hydrogel can be a suitable platform for incorporation of nanotubes and further utilization for environmental applications. In this regard, it is important to compare the absorbance properties of MWCNT in their native form and inside DNA–MWCNT hybrid hydrogel. On the other hand, it is important to characterize the surface state of MWCNT before and after DNA adsorption which can provide the information about the degree of surface coverage by DNA and accessibility of MWCNT surface to other binders.

**Figure 5:**
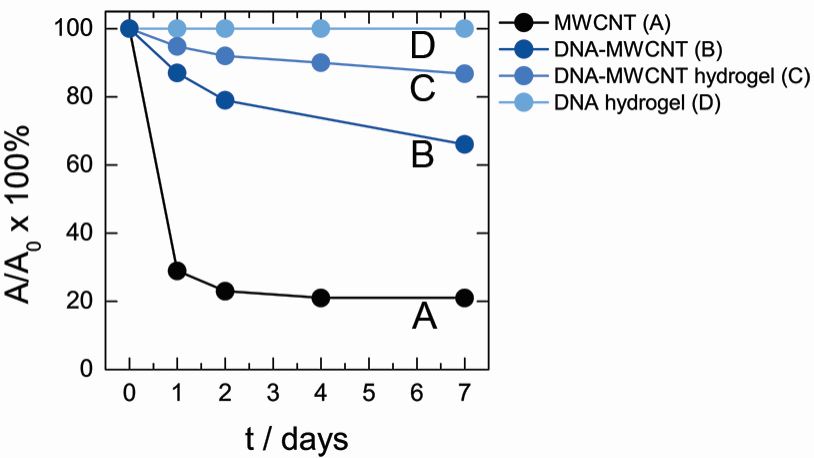
Time-dependent uptake of 1-naphtylamine from 7 mL of 20 μM solution by bare MWCNT (**A**); dispersion of carbon nanotubes in DNA solutions (**B**); DNA–MWCNT hydrogel (**C**); and DNA hydrogel (**D**). The amount of MWCNT in every sample was 1 mg.

DNA has an intrinsic affinity to many aromatic organic chemicals interacting with DNA by groove binding and intercalation mechanisms and this interaction can interfere in the detection of organic chemicals adsorbed by MWCNT. In our recent study (unpublished results) it was found that DNA shows a very low affinity of 1-naphthylamine, therefore, we studied the adsorption of this compound by MWCNT in solution and in hydrogel. Figure 5 compares the percentage of 1-naphtylamine adsorbed by DNA–MWCNT hybrid hydrogel (Figure 5C) and by the same amount of MWCNT in water (Figure 5A) based on spectroscopic measurements of 1-naphtylamine absorbance. Figure 5 shows that although in both cases the concentration of organic component in solution decreased with time, incorporation of nanotubes in DNA hydrogel matrix is accompanied by a significant decrease of MWCNT adsorption capacity by about 5-fold. This decrease can be attributed to either change of MWCNT surface properties due to DNA adsorption or due to localization of MWCNT inside hydrogel. To clarify the mechanism of 1-naphtylamine adsorption inhibition in hydrogel, the adsorption characteristics of MWCNT dispersed by DNA in aqueous solution (Figure 5B) were also evaluated. It was found that the percentage of 1-naphtylamine uptake by MWCNT dispersed by DNA was also lower than that of bare MWCNT but higher than in case of DNA–MWCNT hybrid hydrogel. Based on these findings it can be concluded that the decrease of adsorption properties occurred due to both factors: the passivation of MWCNT surface after DNA adsorption and embedment of MWCNT in the hydrogel.

The time dependence of 1-naphtylamine uptake by MWCNT complexed with DNA (**B** and **C**) is very different from the kinetic adsorption curve of bare MWCNT (**A**). In the latter case, the adsorption of 1-naphtylamine was finished during the first day and no change in 1-naphtylamine concentration above MWCNT was observed later. In contrast, the decrease of 1-naphtylamine concentration in solution with DNA–MWCNT complex or DNA–MWCNT hydrogel continued even after 7 days. Slow uptake kinetics can be attributed to the process of slow exchange of DNA adsorbed on MWCNT with 1-naphtylamine molecules as well as rearrangement of DNA chains.

Data in Figure 5 can be used to roughly estimate the percentage of MWCNT surface coverage by DNA by comparison 1-naphtylamine uptake by pure nanotubes and the uptake by DNA–MWCNT complex and DNA–MWCNT hydrogel. This gives *ca.* 40% coverage for DNA–MWCNT complexes in dispersion and *ca.* 20% for MWCNT in hydrogel, *i.e.*, even after DNA adsorption on MWCNT surface, a substantial part of the surface remains accessible for interaction with low-molecular-weight binders.

Results in Figure 5 indicate that pure MWCNT is more efficient sorbent for organic molecules in comparison to MWCNT embedded in the hydrogel. However, the application of MWCNT in a form of dispersion for environmental cleaning purposes is not possible due to certain issues related to nanotubes’ toxicity and high mobility in the environment. Therefore, the important merit to use MWCNT incorporated in hydrogel is to provide a stable permeable for water matrix incorporating and stabilizing nanotubes inside it. While such nanomaterials can be used directly for environmental cleaning application without concern of environmental pollution, further developments toward better accessibility of nanotube surface are required.

## 3. Experimental Section

### 3.1. Materials

Sodium salt of high molecular weight DNA (*ca.* 20,000 bp) and low molecular weight DNA (300 bp) was a gift from Maruha Nichiro Holdings, Inc. (Tokyo, Japan). MWCNT (diameter 9.5 nm, average length 1.5 μm, 90% purity) was purchased from Nanocyl (Sambreville, Belgium). 1-naphthylamine and *N*,*N*,*N*,*N*-tetramethyl-ethylenediamine (TEMED) from Sigma-Aldrich, Inc. (St. Louis, MO, USA); ethylene glycol diglycidyl ether (EGDE) was from TCI (Tokyo, Japan); sodium borohydride (NaBH_4_) from Sigma-Aldrich; *p*-nitrophenol, sodium hydroxide (NaOH), and sodium chloride (NaCl) from Wako Pure Chemical Industries, Ltd. (Osaka, Japan) were used as received. MilliQ water purified by Simplicity UV apparatus (Merck KGaA, Darmstadt, Germany) was used in all experiments.

### 3.2. Methods

UV-vis spectra of MWCNT were recorded on a Jasco V-630 (Tokyo, Japan) spectrophotometer in 1 mL quartz cells at room temperature. TEM observations were performed at room temperature on a HITACHI H-800 microscope (Tokyo, Japan) at 200 kV acceleration voltage. A 3 mm copper grid covered with a collodion film was placed on a drop of dispersed MWCNT solution. After 5 min, the blotted solution was removed with filter paper, and the sample was dried at room temperature before observation. Dynamic light scattering and zeta potential measurements of MWCNT dispersed by DNA were performed on a Zetasizer Nano ZS (Malvern Instruments Ltd., Worcestershire, UK) at room temperature.

### 3.3. Dispersion of Carbon Nanotubes in DNA Solutions

Solution of DNA was prepared by stirring 1% salmon sperm DNA (*ca.* 300 bp) in 1 mM NaCl solution overnight. MWCNT (1 mg) was incubated with 20 μL of MilliQ water over 24 h. Next, 1 mg of moisten nanotubes was added to 1% solution of DNA (1 mL), 1 mM NaOH solution (10 μL) and 2 mL MilliQ water, mixed by Vortex and sonicated at 10 W (VP-5S, TAITEC, Tokyo, Japan) for 2.5 h. The insoluble residue was removed by centrifugation at 11,000 rpm for 30 min and the resulted solution of dispersed MWCNT was stored in refrigerator (4 °C) and used for hybrid hydrogel preparation as is.

### 3.4. Preparation of DNA Hydrogel

0.05 g of DNA from salmon milt (20,000 bp, purity > 90%) was dissolved in 10 mL of 1 mM NaCl aqueous solution by stirring at room temperature at 300 rpm over 48 h. The resulted 0.5% DNA stock solution was stored in a refrigerator.

To 5 mL of 0.5% DNA solution 15 μL of ethylene glycol diglycidyl ether (EGDE), 50 μL of 0.5 M NaOH, and 5 μL of *N*,*N*,*N*,*N*-tetramethyl-ethylenediamine (TEMED) were subsequently added and mixed by Vortex mixer. The resulted viscous liquid was transferred onto 42 mm Petri dish. Next, the dish was placed on a hot plate, and heated at 90 °C for 20 min covered with upper dish, and then at 50 °C for 2 h without cover dish under ambient conditions. After heating, the reaction mixture was incubated for 12 h at room temperature. The resulted hydrogel was allowed to swell in 1 mM NaCl solution and was then repeatedly washed by 1 mM NaCl solution and stored under the same ionic strength.

### 3.5. Preparation of Hybrid DNA–MWCNT Hydrogel

A quantity of 0.1 g of DNA from salmon milt (20,000 bp) was dissolved in 10 mL of 1 mM NaCl aqueous solution by stirring at room temperature at 300 rpm over 48 h. The resulted 1.0% DNA stock solution was stored in a refrigerator.

A quantity of 2.5 mL of 1% DNA solution was mixes by Vortex with 2.5 mL of MWCNT solution containing various concentrations of DNA of MWCNT prepared in 3.3. Next, to 5 mL of the resulted mixture 15 μL of EGDE, 50 μL of 0.5 M NaOH, and 5 μL of TEMED were subsequently added and mixed by Vortex mixer. The resulted viscous liquid was transferred onto 42 mm Petri dish. Next, the dish was placed on a hot plate, and heated at 90 °C for 20 min covered with upper dish, and then at 50 °C for 2 h without cover dish under ambient conditions. After heating, the reaction mixture was incubated for 12 h at room temperature, the resulted hydrogel was allowed to swell in 1 mM NaCl solution and was then repeatedly washed by 1 mM NaCl solution and stored under the same ionic strength.

## 4. Conclusions

A simple, efficient method for preparation of DNA–MWCNT hybrid hydrogel is reported. In this method, utilization DNA is particularly suitable because it is used for both (i) efficient MWCNT dispersion in aqueous solutions; and (ii) cross-linking between MWCNT–DNA complexes and high molecular weight free DNA. MWCNT can be evenly distributed inside hydrogel, but there exists a critical concentration of nanotubes above which their aggregation of MWCNT inside hydrogel takes place. Incorporation of MWCNT significantly strengthens the mechanical properties of DNA hydrogel as revealed by its shrinking in solutions with high salt concentration. As a result of the passivation of MWCNT surface due to DNA adsorption, the uptake efficiency of organic chemicals by MWCNTs significantly decreased. DNA provides biocompatibility to the hybrid hydrogel and being strengthened by carbon nanotubes it is promising for future applications in tissue engineering [18]. Finally, the utilization of DNA extracted from salmon milt, the waste product of fishery industry, renders the proposed protocol green, sustainable, and suitable for large scale production of the hydrogels.
